# Role of Neuroimaging as a Biomarker for Neurodegenerative Diseases

**DOI:** 10.3389/fneur.2018.00265

**Published:** 2018-04-18

**Authors:** Soichiro Shimizu, Daisuke Hirose, Hirokuni Hatanaka, Naoto Takenoshita, Yoshitsugu Kaneko, Yusuke Ogawa, Hirofumi Sakurai, Haruo Hanyu

**Affiliations:** Department of Geriatric Medicine, Tokyo Medical University, Tokyo, Japan

**Keywords:** amyloid, biomarker, DAT, dementia, MIBG, neuroimaging, tau

## Abstract

It has recently been recognized that neurodegenerative diseases are caused by common cellular and molecular mechanisms including protein aggregation and inclusion body formation. Each type of neurodegenerative disease is characterized by the specific protein that aggregates. In these days, the pathway involved in protein aggregation has been elucidated. These are leading to approaches toward disease-modifying therapies. Neurodegenerative diseases are fundamentally diagnosed pathologically. Therefore, autopsy is essential for a definitive diagnosis of a neurodegenerative disease. However, recently, the development of various molecular brain imaging techniques have enabled pathological changes in the brain to be inferred even without autopsy. Some molecular imaging techniques are described as biomarker in diagnostic criteria of neurodegenerative disease. Magnetic resonance imaging (MRI), single photon emission computed tomography (SPECT), positron emission tomography (PET), and amyloid imaging are described in the diagnostic guidelines for Alzheimer’s disease in the National Institute on Aging-Alzheimer’s Association. MRI, dopamine transporter (DAT) imaging, and ^123^I-metaiodobenzyl-guanidine (MIBG) myocardial scintigraphy listed in the guidelines for consensus clinical diagnostic criteria for dementia with Lewy bodies are described as potential biomarkers. The Movement Disorder Society Progressive Supranuclear Palsy Study Group defined MRI, SPECT/PET, DAT imaging, and tau imaging as biomarkers. Other diagnostic criteria for neurodegenerative disease described neuroimaging findings as only characteristic finding, not as biomarker. In this review, we describe the role of neuroimaging as a potential biomarker for neurodegenerative diseases.

## Introduction

Each neurodegenerative disease type is characterized by the specific protein that aggregates. Recently, extensive research has been performed on disease-modifying therapies for neurodegenerative diseases (i.e., Alzheimer’s disease, tauopathies, etc.), which are expected to be developed in the near future. Simple and practical biomarkers specific for each neurodegenerative disease are urgently required for their accurate diagnosis and facilitate the development of disease-modifying interventions. A recent study demonstrated the potential clinical utility of plasma biomarkers in predicting brain amyloid-β burden (1). However, in daily clinical setting, plasma biomarkers are not available yet and analyzing the cerebrospinal fluid (CSF) of all patients may be difficult (2–4).

In many diagnostic criteria for neurodegenerative disease, characteristic findings in neuroimaging are mentioned. Moreover, only few recent diagnostic criteria for neurodegenerative diseases have mentioned neuroimaging techniques as biomarkers that can estimate pathological changes occurring in the brains of neurodegenerative disease patients (2, 3, 5, 6).

The current mini-review addresses the roles of neuroimaging techniques described as biomarkers on diagnostic criteria for individual neurodegenerative diseases as research topics. Even though there were numerical findings of neuroimaging technique of neurodegenerative disease, we omitted those of not described in diagnostic criteria. For example, about prion disease, hyperintensity in cortex and basal ganglia on FLAIR and DWI are widely known as characteristic magnetic resonance imaging (MRI) finding of prion disease in daily clinical setting. However, this characteristic MRI finding is not described in diagnostic criteria for prion disease which is widely used (7).

In this review, we defined the characteristic neuroimaging findings described in diagnostic criteria from Level A and B based on evidence level as follows:

Level A: able to use the research criteria; described as biomarkers in diagnostic criteria.
Level B: supportive biomarker of clinical diagnosis; described as supportive biomarkers for research criteria in diagnostic criteria.
Level C: supportive of clinical diagnosis; not described as biomarkers but characteristic finding in diagnostic criteria.

Table 1 shows a summary of neuroimaging techniques used as biomarkers, as described in various diagnostic criteria.

**Table 1:** Summary of neuroimaging techniques used as biomarkers.

Imaging technique	Disease	Evidence level	Actual description in diagnostic criteria
MRI	AD	A	Atrophy in temporal lobe sand medial parietal cortex
	DLB	B	Absence of or minimal medial temporal lobe atrophy
	PSP^a^	A–C	Characteristic image findings described for each subtype
SPECT/PET	AD	A	Hypometabolism in temporoparietal cortex
	DLB	B	Hypoperfusion/metabolism in occipital lobe and posterior cingulate island sign on FDG-PET
	PSP^a^	A–C	Frontal lobe hypoperfusion, frontal lobe and midbrain hypometabolism, and frontal hypometabolism
DAT imaging	DLB	A	Reduced dopamine transporter uptake in basal ganglia
	PSP^a^	A–C	Reduced striatal DAT/D2 receptor and reduced brain stem DAT
MIBG	DLB	A	Abnormal (low uptake) on MIBG myocardial scintigraphy
Amyloid PET	AD	A	Positive PET amyloid imaging
Tau PET	PSP^a^	B	THK5351 uptake in midbrain and globus pallidus[^18^F]AV-1451 uptake in midbrain, thalamus, basal ganglia, and dentate nucleus of the cerebellum

*^a^Not described in diagnostic criteria, but defined as a biomarker by the MDS-endorsed PSP Study Group*.

*Evidence levels are different depend on clinical subtypes*.

*MRI, magnetic resonance imaging; SPECT, single photon emission computed tomography; PET, positron emission tomography; DAT imaging, dopamine transporter imaging; MIBG, ^123^iodine-metaiodobenzylguanidine myocardial scintigraphy; AD, Alzheimer’s disease; DLB, dementia with Lewy bodies; PSP, progressive supranuclear palsy; MDS, movement disorder society; FDG, ^18^fluorodeoxyglucose*.

*Level A: able to use the research criteria; described as biomarkers in diagnostic criteria*.

*Level B: supportive biomarker of clinical diagnosis; described as supportive biomarkers for research criteria in diagnostic criteria*.

*Level C: supportive of clinical diagnosis; not described as biomarkers but characteristic finding in diagnostic criteria*.

### Magnetic Resonance Imaging

Magnetic resonance imaging is one of the most widely used neuroimaging techniques for the diagnosis of neurodegenerative diseases. However, among the diagnostic criteria for neurodegenerative diseases, only two diagnostic guidelines, namely, for Alzheimer disease (AD) (2, 3) and for dementia with Lewy bodies (DLB) (5) state characteristic MRI findings as biomarkers.

The National Institute on Aging-Alzheimer’s Association (NIA-AA) diagnostic guidelines for AD state disproportionate atrophy on structural MRI in the medial, basal, and lateral temporal lobes, as well as the medial parietal cortex, as a biomarker of neuronal degeneration or injury (2, 3) (Level A).

The latest diagnostic guidelines of DLB (5) state the absence of or minimal atrophy of the medial temporal lobe on MRI as a supportive biomarker that is consistent with DLB, which assists in the diagnostic evaluation, but does not have clear diagnostic specificity (Level B). Hippocampus is strongly correlated at autopsy with tangles rather than plaque or Lewy body-associated pathology (8). Many studies reported the coexistence of DLB and AD pathology in patients. Most patients with DLB also demonstrate AD pathology (5, 9). Therefore, it should be emphasized that if patients have medial temporal lobe atrophy, DLB should not be denied.

The movement disorder society (MDS) clinical diagnostic criteria for progressive supranuclear palsy (PSP) that has recently been established state a characteristic MRI finding, namely, predominant midbrain atrophy relative to the pons, as a supportive feature, but not a biomarker of PSP (10) (Level C). On the other hand, the MDS-endorsed PSP Study Group classified various neuroimaging techniques into five classes of biomarkers for Richardson’s syndrome (PSP-RS) and the other variant syndromes of PSP (vPSP), which were defined by evidence levels. However, to our knowledge, the top two level evidence biomarkers (i.e., 5: definitive and 4: supportive of pathological diagnosis) have not been described in the literature. On the other hand, characteristic MRI findings on PSP are described in detail as classified biomarkers (level 1: research tool, level 2: supportive of clinical diagnosis, and level 3: supportive of early clinical diagnosis) for individual PSP clinical subtypes (Levels A–C). Although details have been omitted from this review, basal ganglia and thalamic atrophy, and rates of whole-brain and midbrain atrophy are mentioned as representative examples of biomarkers for PSP-RS, and midbrain atrophy is mentioned as a biomarker of vPSP. Furthermore, it is noteworthy that not only structural MRI findings but also findings displayed on functional MRI and diffusion tensor imaging are described. Details of the MRI findings are described elsewhere (6).

Other diagnostic criteria for neurodegenerative diseases also describe characteristic findings on MRI, but there are no descriptions of biomarkers. Frontotemporal lobar degeneration (FTLD) has three clinical subtypes (11), which demonstrate various pathological changes (e.g., FTLD-tau, FTLD-TDP, FTLD-UPS, and FTLD-FUS FTLD-ni) (12). The diagnostic criteria for FTLD that is most commonly used (11) mentions MRI findings of individual clinical subtypes as characteristics, rather than as biomarkers (Level C). As FTLD is caused by various pathological changes, it is difficult to consider neuroimaging data, including those from MRI, as biomarkers. Recent diagnostic criteria for the behavioral variant of frontotemporal dementia (bvFTD) (13) mentioned frontal or anterior temporal atrophy displayed on MRI or computed tomography as characteristic findings suggesting a diagnosis of bvFTD.

A second consensus statement on the diagnosis of multiple system atrophy (MSA) mentioned atrophy displayed on MRI of the putamen, middle cerebellar peduncle, pons, or cerebellum as an additional feature suggestive of MSA-P or MSA-C, but not as a biomarker (14) (Level C).

However, many past studies showed, coexistence of several pathological changes is not rare in elderly patients (5, 9, 15). Therefore, existence of individual pathological change is suggested due to the above MRI characteristic findings, however, it should be noted that coexistence of other pathological changes cannot be denied.

### Single Photon Emission Computed Tomography (SPECT) and Positron Emission Tomography (PET)

Recently, among the nuclear medicine imaging techniques, SPECT and PET have been used for the diagnosis of neurodegenerative diseases in the daily clinical setting and are the most commonly used new neuroimaging techniques.

The NIA-AA diagnostic guidelines for AD describe decreased ^18^fluorodeoxyglucose (FDG) uptake on PET in the temporoparietal cortex as a biomarker of neuronal degeneration or injury (2, 3) (Level A).

The most recent diagnostic criteria for DLB (5) mentioned hypoperfusion/hypometabolism by SPECT/PET in the occipital lobe and the posterior cingulate island sign on FDG-PET imaging as supportive biomarkers (Level B). An autopsy-confirmed study suggested that FDG-PET occipital hypometabolism was able to differentiate DLB from AD with high accuracy (16). Larger studies on patients earlier in the course of the disease suggested a sensitivity (70%) and specificity (74%) slightly lower than required for an indicative biomarker, although better than that reported for SPECT (65 and 64%, respectively) (17, 18). On the other hand, our past study showed that there was no significant perfusion differences in medial occipital lobe between AD and DLB (19). In any case, compared with dopamine transporter (DAT) imaging ^123^Iodine-metaiodobenzylguanidine (MIBG) cardiac scintigraphy described below, occipital hypoperfusion has low sensitivity and specificity. Therefore, it is understandable that it stayed in only one of the supportive biomarker with most resent diagnostic criteria. Relative preservation of the metabolic activities of the posterior cingulate cortex and midcingulate cortex on FDG-PET (the cingulate island sign) has been described in DLB (20), associated with less concurrent neurofibrillary pathology, but with no difference in amyloid-beta (Aβ) load relative to AD (21).

In the recent MDS clinical diagnostic criteria for PSP, predominant midbrain hypometabolism relative to the pons displayed on FDG-PET is described as a supportive feature, rather than a biomarker (10) (Level C). On the other hand, the MDS PSP Study Group defined SPECT frontal hypoperfusion as a level 1 biomarker (research tool), FDG-PET frontal and midbrain hypometabolism as a level 2 biomarker (supportive of clinical diagnosis), and FDG-PET frontal hypometabolism as a class 3 biomarker (supportive of early clinical diagnosis) for PSP-RS (6) (Levels A–C).

Recent diagnostic criteria for bvFTD (13) stated frontal or anterior temporal hypoperfusion or hypometabolism displayed on PET or SPECT as characteristic neuroimaging data suggestive of bvFTD (Level C). This criteria states that functional imaging changes may provide additional sensitivity, suggesting that behavioral and functional abnormalities may precede structural imaging changes in bvFTD.

The second consensus statement on MSA stated hypometabolism on FDG-PET in the putamen, brainstem, and cerebellum as additional features of possible MSA-P and hypometabolism on FDG-PET in the putamen as an additional feature of possible MSA-C, rather than as biomarkers (14) (Level C).

### Dopamine Transporter Imaging

DAT imaging use a ligand that binds to the presynaptic dopamine transporter, can be used to analyze the integrity of the nigrostriatal projection pathway. ^123^I-2β-carbomethoxy-3β-(4-iodophenyl)-*N*-(3-fluoropropyl) nortropane (^123^I-FP-CIT) is the ligand for DAT-SPECT that is most widely used. ^123^I-FP-CIT has been used in a large number of trials to identify the *in vivo* loss of dopamine transporters in the striatum of patients with presynaptic parkinsonism (22, 23). Therefore, Parkinson syndrome including Parkinson’s disease (PD) that has the dysfunction of the nigrostriatal projection pathway showed reduced DAT uptake. In other words, DAT imaging is not suitable for differentiation of presynaptic Parkinson syndrome. Therefore, the recent clinical diagnostic criteria for PD by the MDS did not mention DAT imaging as biomarker or characteristic imaging finding of PD (24). DAT imaging requires attention to use in the following cases, patients with an infarction in the basal ganglia, patients who are unable to stop the use of medications that affect DAT uptake (e.g., cocaine, amphetamines, bupropion, selective serotonin reuptake inhibitors, etc.) (25).

However, DAT imaging is useful for differentiating DLB from other dementias. The latest diagnostic guidelines of DLB state reduced dopamine transporter (DAT) uptake in the basal ganglia displayed on SPECT or PET as indicative biomarkers (5) (Level A). However, there are no biomarkers available for the clinical diagnosis of Lewy body-associated pathology. DAT imaging was described as a useful indirect method for determining Lewy body-associated pathology. Reduced DAT uptake in the basal ganglia has been confirmed by SPECT or PET imaging. The utility of DAT imaging in distinguishing DLB from AD is well established, with a sensitivity of 78% and a specificity of 90% (26).

In the recent MDS clinical diagnostic criteria for PSP, postsynaptic striatal dopaminergic degeneration, as demonstrated for example by ^123^I-iodobenzamide (IBZM)-SPECT or ^18^F-desmethoxyfallypride (DMFP)-PET, is described as a supportive feature rather than as a biomarker (10) (Level C). However, the MDS PSP Study Group defined reduced striatal DAT/D2 receptor levels and reduced brainstem DAT levels as level 2 biomarkers for PSP-RS (Level B), and reduced striatal DAT as a level 1 biomarker for some types of vPSP (6) (Level A).

The second consensus statement on the diagnosis of MSA states presynaptic nigrostriatal dopaminergic denervation displayed on SPECT or PET as an additional feature suggestive of MSA-P, but not as a biomarker (14) (Level C).

### ^123^Iodine-Metaiodobenzylguanidine Myocardial Scintigraphy

MIBG is a physiologic analog of noradrenaline used to determine the location, integrity, and function of postganglionic noradrenergic neurons. Lewy body disease including DLB and PD presents an impairment of adrenergic function and consequently an abnormal MIBG myocardial syntigraphy (27). MIBG myocardial scintigraphy requires attention to patients with heart disease, diabetes, or thyroid disease, or patients taking any drugs known to affect the accumulation of MIBG (28).

The latest diagnostic guidelines of DLB state abnormal (low uptake) MIBG myocardial scintigraphy as an indicative biomarker (5) (Level A). MIBG myocardial scintigraphy quantifies postganglionic sympathetic cardiac innervation, which is reduced in Lewy body disease (27, 29). The sensitivity and specificity values of MIBG myocardial scintigraphy for discriminating probable DLB from probable AD rise from 69 and 87%, respectively, in severely demented patients to 77 and 94%, respectively, in mildly demented patients (30).

The recent clinical diagnostic criteria for PD by the MDS described the presence of either olfactory loss or cardiac sympathetic denervation on MIBG myocardial scintigraphy as supportive criteria, but not as a biomarker (24) (Level B).

In MSA, which is an α-synucleinopathy similar to PD, the diagnostic criteria (14) also does not state MIBG myocardial scintigraphy as supportive diagnostic criteria (Level C). The reason is that there is no consensus on the usefulness of MIBG myocardial scintigraphy for diagnosing MSA. Many reports using MIBG myocardial scintigraphy have shown preserved sympathetic postganglionic neurons in MSA, in contrast to in PD (31). On the other hand, some studies have shown denervation in patients with MSA displayed on MIBG myocardial scintigraphy (32).

### Aβ Imaging

Interest in the use of Aβ imaging for the diagnosis of AD has been increasing. Aβ imaging is a tool expected to be new possibilities for the early detection, intervention, and prevention of AD. NIA-AA diagnostic guidelines for AD showed that biomarkers of brain Aβ protein deposition are low CSF Aβ42 and positive PET amyloid imaging (2, 3) (Level A). In addition, the diagnostic criteria do not specify the particular amyloid imaging tracer to be used. Studies with ^11^C-Pittsburgh compound-B (^11^C-PiB), the first and most widely studied PET Aβ ligand, indicated that Aβ imaging may enable the earlier diagnosis of AD (33, 34). However, owing to the 20-min half-life of ^11^C, ^11^C-PiB can only be used in large PET centers with their own on-site cyclotron and radiopharmacy facilities. ^18^F is a more suitable radioisotope for widespread clinical use as its longer half-life of 110 min enables distribution from a production site to multiple PET centers. Recently, ^18^F-labeled tracers have been developed, which are starting to be used clinically. The actual ^18^F-labeled tracers being used are as follows: flutemetamol (GE Healthcare), florbetapir (Amyvid, Eli Lilly), florbetaben (Neuraceq, Piramal Imaging), and ^18^F-AZD4694 (recently renamed NAV4694) (Astra-Zeneca, Navidea) are derived from stilbene. The above ^18^F-labeled tracers except NAV4694 have been approved by the Food and Drug Administration. Correlations have been shown between levels of biomarker CSF Aβ42 and PET signals using ^11^C-PiB as a tracer for Aβdeposits in the brain (35) and comparable studies have been performed for ^18^F-labeled amyloid tracers (36, 37). A systematic review found no marked differences in the diagnostic accuracy among flutemetamol, florbetapir, and florbetaben (38). These three tracers perform better when used to differentiate between patients with AD and healthy controls. Furthermore, a study showed that NAV4694 displays high cortical binding and low nonspecific white matter binding in AD patients (39).

On the other hand, NIA-AA diagnostic guidelines for AD described, “guidelines do not advocate the use of AD biomarker tests for routine diagnostic purposes at the present time” (2, 3). At the present time, amyloid imaging should be used for research criteria of AD, taking into consideration that it is not possible at all facilities. Further studies using amyloid imaging are required to identify the tracer with the highest sensitivity and specificity, and to identify the positioning of Aβ imaging in clinical use. However, we believe that amyloid imaging, particularly ^18^F-labeled tracers, contribute to the early diagnosis of AD.

### Tau Imaging

The advances of molecular imaging in recent years have led to the development of promising tau-specific PET tracers. In recent years, hot topics were shifted from general neuroimaging to molecular imaging of the neurodegeneration related to tau protein (40).

Unfortunately, tau imaging has not been stated as a biomarker in any diagnostic criteria for neurodegenerative diseases. However, the MDS PSP study group defined two tau-specific tracers (i.e., ^18^F-THK5351 and ^18^F-AV1451) as a biomarker of PSP-RS (6) (Level B). Because very few articles have been published on this subject, it has been suggested to have low reliability.

However, tau imaging is expected to become an important biomarker of tau pathology in the future. There are six different isoforms of tau, formed by alternative mRNA splicing of the microtubule-associated protein tau (*MAPT*) gene. More importantly, the inclusion or exclusion of exon 10 results in either 4 repeats (4R) or 3 repeats (3R) of the microtubule-binding domain being transcribed in the tau protein, respectively (41). The 3R/4R ratio is 1:1 under physiological conditions and in patients with AD, tangle predominant senile dementia, and chronic traumatic encephalopathy. 3R isoforms are dominant in FTD and 4R isoforms are dominant in corticobasal degeneration, PSP, and argyrophilic grain disease (42).

The radiotracers used to image tau neurofibrillary tangle deposition in living AD patient brains are ^18^F-FDDNP (43), ^18^F-AV1451 (44), ^11^C-PBB3 (45), and ^18^F-THK5351 (46). These tracers are now available for clinical assessment of patients with various tauopathies, including AD, as well as in healthy subjects.

However, PET tracers used for tau imaging still have some problems that remain to be resolved. These tracers often show “off-target” binding. Recently, it has become clear that these tracers detected the distribution of not only tau but also other proteins (47). For example, ^18^F-THK5351 has been confirmed to also bind to monoamine oxidase B (48). Moreover, ^18^F-AV1451 is well known to bind to calcifications, iron, melanin, and blood vessels (49). Therefore, further studies of a large number of patients, with consideration of the results of pathological analyses are required. Even considering the above issues, we believe that with further research, tau imaging will become a useful biomarker in the near future.

## Conclusion

In this review, we introduced the various neuroimaging techniques described in the current diagnostic criteria for neurodegenerative diseases and the possibility of new neuroimaging techniques as biomarkers. We believe that further advances in these neuroimaging techniques will lead to useful biomarkers that can accurately predict the pathological changes occurring in various neurodegenerative diseases.

## Ethics Statement

All procedures performed in the studies involving human participants were in accordance with the ethical standards of the institutional or national research committee and with the 1964 Helsinki declaration and its later amendments or comparable ethical standards. Informed consent: informed consent was obtained from all individual participants included in the study.

## Author Contributions

SS, DH, Hi.H, NT, YK, and YO wrote the manuscript. HS and Ha.H critically reviewed the manuscript.

## Conflict of Interest Statement

The authors declare that the research was conducted in the absence of any commercial or financial relationships that could be construed as a potential conflict of interest.
